# *Solanummedusae* (Solanaceae), a new wolf-fruit from Brazil, and a key to the extra-Amazonian Brazilian Androceras/Crinitum Clade species

**DOI:** 10.3897/phytokeys.118.31598

**Published:** 2019-02-27

**Authors:** Yuri Fernandes Gouvêa, João Renato tehmann, Sandra Knapp

**Affiliations:** 1 Departamento de Botânica, Instituto de Ciências Biológicas, Universidade Federal de Minas Gerais – UFMG, Av. Antônio Carlos, 6627, Pampulha, Belo Horizonte, CEP 31270-901, MG, Brazil Universidade Federal de Minas Gerais Belo Horizonte Brazil; 2 Department of Life Sciences, Natural History Museum, Cromwell Road, London SW7 5BD, UK The Natural History Museum London United Kingdom

**Keywords:** Brazil, Cerrado, new species, wolf-fruit, identification key, prickly *Solanum*, Solanaceae

## Abstract

*Solanummedusae***sp. nov.** is described from the Cerrado biome in the Serra da Canastra region, southwestern Minas Gerais State, Brazil. The new species is morphologically similar to the common *S.lycocarpum* A.St.-Hil. (known as lobeira or wolf-fruit), but differs from it in habit and pubescence characters. We here describe this new taxon and discuss its morphology, some aspects of its ecology, affinities and distribution. Full specimen citations are provided, as well as illustrations, distribution map and a preliminary conservation assessment of the species. A key to all of the known extra-Amazonian Brazilian species of the Androceras/Crinitum clade is also provided to aid in their identification.

## Abstract (es)

*Solanummedusae***sp. nov.** é descrita para o Cerrado da região da Serra da Canastra, sudoeste do estado de Minas Gerais, Brasil. A nova espécie é morfologicamente semelhante à comum *S.lycocarpum* A.St.-Hil. (conhecida como lobeira ou fruta-do-lobo), da qual pode ser diferenciada por características do hábito e do indumento. O presente trabalho descreve este novo táxon, discute sua morfologia, alguns aspectos da sua ecologia, suas afinidades e distribuição. Citações completas dos espécimes são fornecidas, assim como ilustrações, mapa de distribuição e uma avaliação preliminar do estado de conservação da espécie. Uma chave de identificação para todas as espécies conhecidas do clado Androceras/Crinitum ocorrentes no Brasil que possuem distribuição extra-amazônica também é fornecida.

## Introduction

*Solanum* L. (Solanaceae) is the largest genus of Solanaceae, with some 1,400 species, and one of the biggest angiosperm genera (Frodin 2004). Occurring on all temperate and tropical continents, *Solanum* has its highest diversity of both clades and species in tropical South America, the region considered to be ancestral for the family and major clades within it (Dupin et al. 2017). The last complete monograph of the genus was De Candolle’s "*Prodromus*" (Dunal 1852), which included 901 species (with an additional 19 incompletely known). *Solanum* taxonomy has proceeded in a piecemeal fashion until relatively recently and the genus had acquired a reputation of being intractable, but recent monographic work has begun to remedy this situation.

The largest monophyletic group of *Solanum*, known as the Leptostemonum clade or SolanumsubgenusLeptostemonum Bitter (Bohs 2005; Weese and Bohs 2007), includes prickly plants with stellate indumentum (the “spiny” solanums) and comprises approximately half the species diversity of the genus. It is composed of a large lineage of approximately 240 species confined to the Old World (see Aubriot et al. 2016), with the remainder primarily New World in distribution. Brazil is a center of diversity for all groups of *Solanum* (see Knapp et al. 2015), including the spiny solanums. Recent intensive work on the Brazilian flora for the Flora do Brasil project (BFG 2015, 2018) has resulted in better understanding of species diversity and distribution in *Solanum*, but numerous new taxa continue to be discovered in the country (e.g., Giacomin and Stehmann 2014; Agra and Stehmann 2016; Gouvêa and Stehmann 2016; Stehmann and Moreira 2016), even in southeastern Brazil, considered to be the best-explored region of the country (Sobral and Stehmann 2009; Forzza et al. 2012; Sousa-Baena et al. 2014).

The recent discovery of *Solanum* species from places close to urban centers where the flora would be expected to be well-known (e.g., Agra and Stehmann 2016; Stehmann and Moreira 2016; Gouvêa and Stehmann 2016; Gouvêa et al. 2018) coupled with threats to tropical vegetation in general draws attention to the continued urgency and relevance of efforts aimed at describing and preserving the still insufficiently known diversity of the Brazilian flora.

Here we describe a new species related to *S.lycocarpum* A.St.-Hil. (Androceras/Crinitum clade sensu Levin et al. 2006; Stern et al. 2011), an iconic species from Brazilian Cerrado (savannah-like vegetation), discuss its morphology, conservation status, distribution and affinities, and present a key to the extra-Amazonian Brazilian species of the group.

## Materials and methods

Following discovery of the new species two expeditions were carried out to the Serra da Canastra (Apr 2017 and May 2018) in order to increase our sampling and to ascertain the distribution of *S.medusae*. Specimens with coordinates were mapped directly, and all specimens are cited in the Specimens examined portion of the text. Descriptions are based on field work of YFG and examination of herbarium specimens. Specimens were examined from ALCB, BHCB, CEPEC, HUEFS, HUFU, R, RB, UEC, and UB (acronyms follow Index Herbariorum; http://sweetgum.nybg.org/science/ih/); online specimens from HUFU were also examined (Reflora - Herbário Virtual: http://reflora.jbrj.gov.br/reflora/herbarioVirtual/). Measurements of reproductive characters were taken from both fresh and dried material. Trichome types follow the terminology proposed in Roe (1972) and Mentz et al. (2000), while that of the general morphology is mainly based on Radford et al. (1976).

Extent of Occurrence (EOO) and Area of Occupancy (AOO) were calculated using GeoCat (www.geocat.kew.org) with a 2 km cell width for AOO calculation. The preliminary conservation status was assessed using the IUCN (2017) criteria based on the GeoCat analyses (Bachman et al. 2011) combined with field knowledge. All specimens examined are cited in the text. Our delimitation of *S.medusae* as it is here presented was based on the “morphological species concept” (Davis and Heywood 1963; Mallet 1995).

## Taxonomic treatment

### Solanum medusae

Taxon classificationPlantaeSolanalesSolanaceae

Gouvêa sp. nov.

urn:lsid:ipni.org:names:77195390-1

Figures 1 , 2 , 3

#### Diagnosis.

Like *Solanumlycocarpum* A.St.-Hil., but differing in its decumbent habit and densely glandular pubescence of stems and leaves.

#### Type.

Brazil. Minas Gerais: Distrito de São Roque de Minas, Parque Nacional da Serra da Canastra, principal estrada de terra que leva de São Roque de Minas à portaria do PN Serra da Canastra (passando por Capão Forro), 20°15'35"S, 46°24'36"W, 1212 m, 5 Apr 2017, *Y.F. Gouvêa, T.E. Almeida, A. Salino & I.O. Moura 230* (holotype (2 sheets): BHCB [BHCB188229 (fl), BHCB188229_2 (fr)]; isotypes: HUFU, RB, UB).

#### Description.

Decumbent, spreading shrub to 1 m tall and 3 m in diameter, strongly armed. Young stems terete, green to deep purple, the epidermis sometimes with a varnished appearance, nearly glabrous to pubescent with porrect short- to long-stalked stellate trichomes, the stalks up to 2 mm long, multiseriate, the rays 6–8(–11), 0.2–0.5 mm long, the midpoints shorter than or equal in length to the rays, glandular or eglandular; the stem surface more densely covered with variously sized simple glandular trichomes; smaller papillae-like glandular trichomes 0.1–0.2 mm long, 1–4-celled, uniseriate, the gland single-celled; and longer glandular trichomes to 0.5(–1.8) mm long, multiseriate at the base with single-celled apical glands; prickles (0.2–)0.5–0.7(–0.9) cm long, orange-yellow, broad-based and strongly curved, the base 1–5 mm wide; new growth densely tomentose to pubescent, prickly, pale beige in color in dried plants; stellate trichomes with multiseriate stalks 0.5–1 mm long, the rays 6–10, ca. 0.5 mm long, the glandular or eglandular midpoint shorter than the rays; simple glandular trichomes denser than the stellate trichomes, the shorter papillae-like ones uniseriate, to 0.2 mm long, and the longer ones to 1.5 mm long, multiseriate at the base; prickles 1–5 mm long, strongly curved, yellow, usually tipped with stellate trichomes and sparsely to densely pubescent on the surface with short- to long-stalked stellate trichomes and simple glandular trichomes; bark of older stems reddish purple in live plants and shiny dark reddish brown in herbarium specimens. Sympodial units difoliate, the leaves not geminate. Leaves simple, shallowly lobed, the blades 9–22 cm long, 3–9.3 cm wide, narrowly ovate or trowel-shaped, widest in the lower third, chartaceous, concolorous, armed on both surfaces with curved yellow prickles 0.1–1 cm long, these denser abaxially; adaxial surface epidermis always visible, usually shiny with a varnished appearance, uniformly and sparsely to moderately pubescent with porrect stellate short- to long-stalked trichomes, the stalk 0.2–0.5 mm long, multiseriate, the rays 6–8(10), 0.2–0.5 mm long, the midpoint shorter than the rays and occasionally glandular, these sometimes more densely distributed near the margins, more densely pubescent with simple uniseriate papillae-like glandular trichomes to 0.2 mm long, and 2–3 celled gland-tipped simple trichomes from a multiseriate base; abaxial surface with the epidermis always visible, usually shiny with a varnished appearance, moderately to densely pubescent with stellate and simple trichomes like those of the adaxial surfaces, but the simple glandular trichomes and papillae denser on the lamina; principal veins 4–8 pairs, the finer venation prominent, pale yellow and visible as a complex net on the abaxial surfaces, prickly with curved yellow prickles; base attenuate to abruptly truncate, obtuse or rounded, if attenuate then decurrent onto the petiole, asymmetric or not; margins shallowly lobed, the lobes (1)3–4 on each side of the midvein, rounded and semi-circular in outline, the sinuses less than 1/3 of the distance to the midvein; apex long acuminate, the ultimate tip somewhat rounded; petiole 0.5–5.5 cm long, pubescent like the stems, armed with prickles like those of the stems. Inflorescences 4.5–12 cm long, internodal, usually unbranched, less frequently furcate, with 4–15 flowers, sparsely to densely stellate-pubescent and densely glandular pubescent with trichomes like the stems, densely and irregularly prickly along the entire axis with curved yellow prickles 0.1–0.7 cm long, peduncle 1–2 cm long, prickly and pubescent; pedicels 0.6–2 cm long, ca. 1–1.5 mm in diameter at base and apex, spreading, sparsely to densely prickly, the prickles ca. 5 mm long, straight, usually denser on the basal flower, but in more pubescent individuals all pedicels prickly, articulated at the base; pedicel scars widely spaced 1–2 cm apart near the base of the inflorescence, more closely spaced distally. Buds long-fusiform and tapering, the corolla included in the fused calyx lobes until just before anthesis. Flowers 5-merous (occasionally 4-merous some flowers), slightly zygomorphic (see discussion), heteromorphic, 1(–3) long-styled hermaphroditic flowers at the base of the inflorescence, more distal flowers short-styled and functionally staminate, the plants andromonoecious. Calyx with the tube ca. 3 mm long, obconical to cupuliform, pubescent like the rest of the inflorescence, densely prickly with straight yellow prickles; the lobes 1.2–2 cm long, foliose, lanceolate to long-triangular, strongly reflexed at anthesis, abaxially pubescent and prickly like the rest of the inflorescence, adaxially pubescent with minute sessile or short-stalked porrect-stellate trichomes to 0.2 mm long, the basal hermaphroditic flower more densely prickly and more distal flower calyces often lacking prickles. Corolla 3–6.5 cm in diameter, deep purple in younger flowers, becoming lilac with flower age, the color deeper adaxially, stellate, lobed ca. halfway to the base, the lobes 0.9–2.5 cm long, 1–2 cm wide, spreading, slightly to strongly reflexed at anthesis, abaxially densely stellate-pubescent where exposed in bud, the interpetalar tissue glabrous, adaxially densely papillate with minute stellate trichomes along the midvein, the tips acuminate, the acumens 3–4 mm long, cucullate and densely stellate-pubescent abaxially. Stamens slightly unequal, the upper 2 slightly shorter than the other 3; filament tube 0.8–1.5 mm long, glabrous; free portion of the filaments 1.4–2.5 mm long, glabrous; anthers 12.5–18.5 mm long, 1.7–2.6 mm wide at the base, strongly tapering, the 3 lower longer anthers more or less curved upward in their distal portion, yellow, poricidal at the tips, the pores distally directed, the connective abaxially pubescent with weak-walled white to deep purple stellate trichomes along the entire length. Ovary globose, densely stellate-pubescent with hyaline eglandular many-rayed trichomes, the rays and midpoints equal and not easily distinguishable; style 15–19 mm long in long-styled flowers, curved upwards, glabrous to moderately brown-stalked stellate-pubescent in the basal half, densely glandular papillate near the apex; stigma capitate to strongly bi-lobed (or sometimes with several irregular lobes), green in live plants, the surface densely papillate. Fruit a globose or depressed-globose berry, 7–15 cm in diameter, green becoming yellowish green and sweetly fragrant when ripe, the pericarp smooth, sparsely pubescent with minute stellate trichomes, especially near the pedicel, the mesocarp spongy, pale cream; fruiting pedicels 1.8–2.5 cm long, 1.1–1.3 cm in diameter at the base, 6.5–8.5 mm in diameter at the apex, fleshy in live plants, woody in dry specimens, strongly deflexed downwards so some fruits rest on the soil; fruiting calyx lobes ca. 2 cm long, persistent, prickly or not. Seeds > 100 per berry, 6–7 mm long, 5–6.2 mm wide, flattened reniform, dark brown to blackish brown, drying gray to dark gray, the surfaces minutely pitted, the testal cells sinuate in outline. Chromosome number not known.

**Figure 1.**
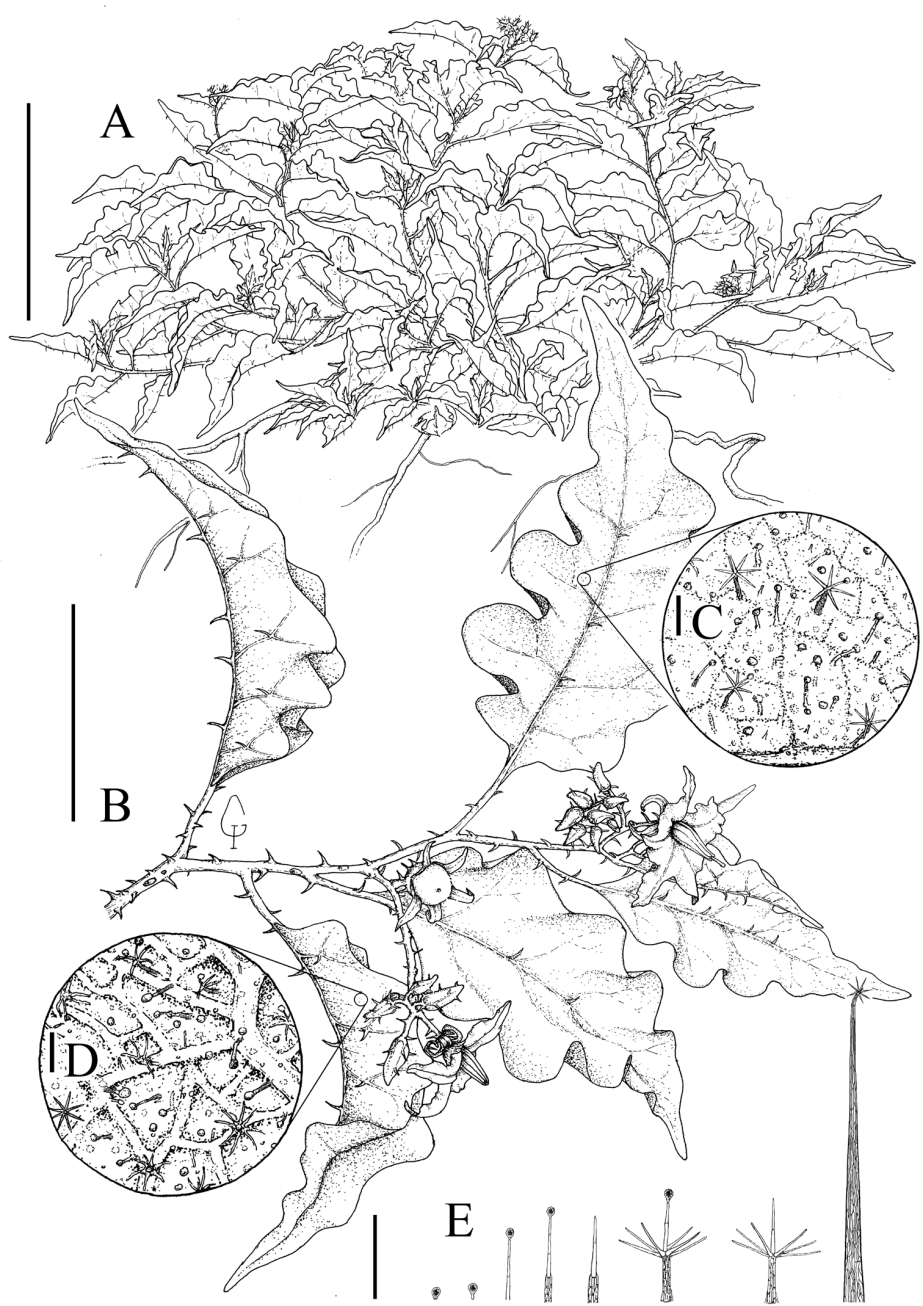
*Solanummedusae.***A** Habit **B** Flowering branch with an immature fruit **C** Detail of the adaxial leaf surface indumentum **D** Detail of the abaxial leaf surface indumentum **E** Trichome types from stems and leaves (*Y.F. Gouvêa et al. 230*, BHCB). Scale bars: 30 cm (**A**), 8 cm (**B**), 0.5 mm (**C–E**). Drawings by Iago F. Gouvêa.

#### Distribution

(Figure 4). *Solanummedusae* is only known from the region of the Serra da Canastra in southwestern Minas Gerais state, Brazil. It has been collected from six municipalities located northeast (Campinópolis, Piumhi, São José do Barreiro and São Roque de Minas), north (São João Batista da Serra) and west (Sacramento) of the Serra da Canastra.

#### Ecology.

**Figure 2.**
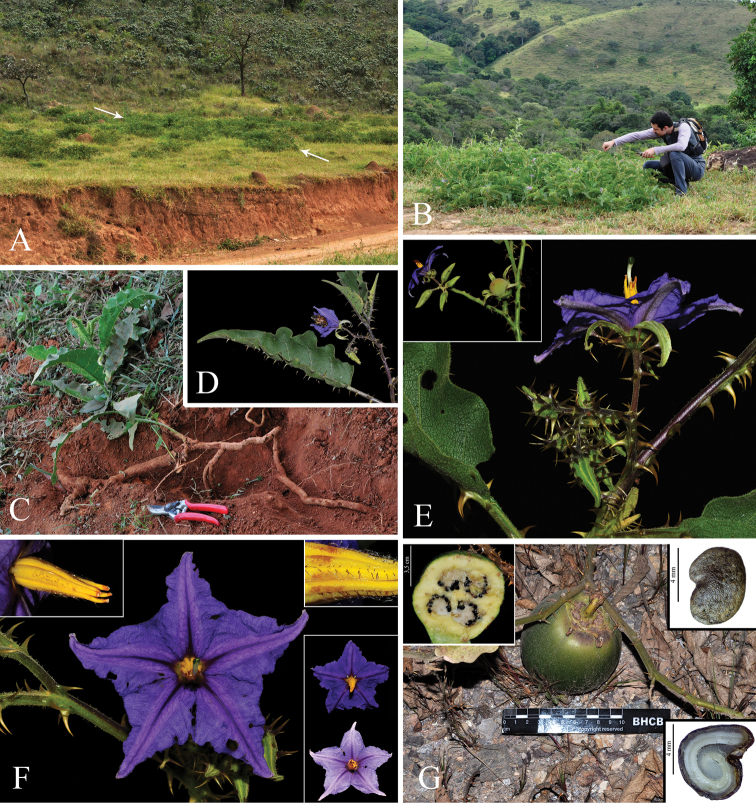
*Solanummedusae*. **A** Habitat **B** Habit; note the distinctive decumbent posture **C** Roots; note the horizontal growth **D** Branch apex; note the deep purple coloration and leaf shape **E** Inflorescence; note that the first flower is always long-styled (upper left corner: a more developed inflorescence with an immature fruit being formed from its first flower, and short-styled flowers distally, some of which have already fallen) **F** Long-styled flower (upper right corner: detail of the slightly unequal anthers with stellate-pubescent connectives; bottom right corner: color difference between the purple post-anthesis corollas and the lilac senescent ones) **G** Fruit (upper left corner: half of a transversally dissected fruit; upper right corner: seed; bottom right corner: dissected embryo). Photographs **A, C–G** by Y.F. Gouvêa **B** by Philipe S. Saviott.

*Solanummedusae* grows in open areas along roads, pastures and clearings in Cerrado, above 700 m elevation (Figure 2A). Populations have been found in areas originally dominated by Cerrado *strictosensu* (lower areas), grasslands (higher areas) and seasonal semi-deciduous tropical forests (mountain slopes).

The poricidal anthers of *S.medusae* (similar to the vast majority of *Solanum* species; Figure 2F) narrow down the spectrum of pollinators to female bees able to collect pollen by vibrating their indirect flight muscles (buzz-pollination; Michener 1962, Buchmann 1983). We observed medium- to large-sized bees (e.g., genera *Ptiloglossa* and *Bombus*; Figure 2D) frequently visiting flowers.

The berries have a suite of characters associated with frugivory and dispersal by terrestrial mammals (Van der Pijl 1972): large size, green to greenish yellow coloration, strong sweet scent released at maturity and close proximity to the soil (Figure 2G). The berries of *S.medusae* are similar to those of *S.lycocarpum* (see Discussion), which have been shown to be a primary plant food of the maned wolf (*Chrysocyonbrachyurus* Illiger, 1811; Santos et al. 2003; Juarez and Marinho-Filho 2002; Jácomo et al. 2004). The known presence of maned wolves in Serra da Canastra (Queirolo and Motta-Junior 2007; Bizerril et al. 2011) suggest that they may act as an important dispersal agent of *S.medusae* as well. Dispersal by maned wolves would also help to explain abundance of *S.medusae* plants along roadsides and other areas where more primary vegetation has been suppressed, since these open places are often used by maned wolves for travel and feeding (Santos et al. 2003; Coelho et al. 2008). Nevertheless, further study is needed to better understand the relationships of *S.medusae* with its putative dispersal and pollination agents.

#### Etymology.

The specific epithet is derived from the snake-like appearance of the prostrate branches and the overall appearance of the habit, resembling the hair of the monster Medusa of Greek mythology.

#### Conservation status

(IUCN 2017). *Solanummedusae* is classified as endangered (EN-B1, B2+bii, iii, ciii, iv) according to the IUCN Red List Categories, based on its relatively restricted extent of occurrence (EOO = 2,146 km^2^ < 5,000 km^2^) and area of occupancy (AOO = 80 km^2^ < 500 km^2^). In addition to its restricted distribution, threats posed by the ongoing agricultural and urban expansion in the region of the Serra da Canastra are high; in this area native Cerrado vegetation has already been replaced by agricultural monocultures (e.g., sugarcane, soybean and coffee) and pastures. The high frequency of non-natural fires in areas of native vegetation also poses a considerable risk. The presence of *S.medusae* in some areas of the Parque Nacional da Serra da Canastra is encouraging, but not enough to eliminate important risks, such as the loss of genetic diversity.

#### Discussion.

*Solanummedusae* belongs to the large monophyletic group commonly known as the spiny solanums (Leptostemonum Clade, sensu Bohs 2005) and is morphologically a member of the “*S.crinitum* group” (sensu Whalen 1984; section Crinitum (Whalen) Child). This group is part of the molecularly defined Androceras/Crinitum clade (sensu Levin et al. 2006; Stern et al. 2011), and includes prickly herbs of the Mexican deserts with dry fruits such as *S.rostratum* Dunal (section Androceras (Nutt.) Whalen, see Whalen 1979), Amazonian vines (i.e. *S.coriaceum* Dunal and *S.sendtnerianum* Van Heurck & Müll.Arg.) and large woody shrubs to trees with relatively large, showy, lilac to deep bluish-purple flowers mostly found in South America (*S.crinitum* group sensu Whalen 1984; Nee 1999). This latter group includes about 15 species (see Whalen 1984; Nee 1999; Farruggia and Bohs 2010; Farruggia et al. 2010), of which at least 10 occur in Brazil; four of these are exclusively Amazonian (i.e., *S.acanthodes* Hook.f., *S.altissimum* Benítez, *S.orientale* Benítez and *S.tricuspidatum* Dunal), and six have strictly or essentially extra-Amazonian distributions (*S.crinitum* Lam., *S.falciforme* Farruggia, *S.gomphodes* Dunal, *S.lycocarpum*, *S.medusae* and *S.quaesitum* C.V.Morton). We present a key for the extra-Amazonian Brazilian species below.

**Figure 3.**
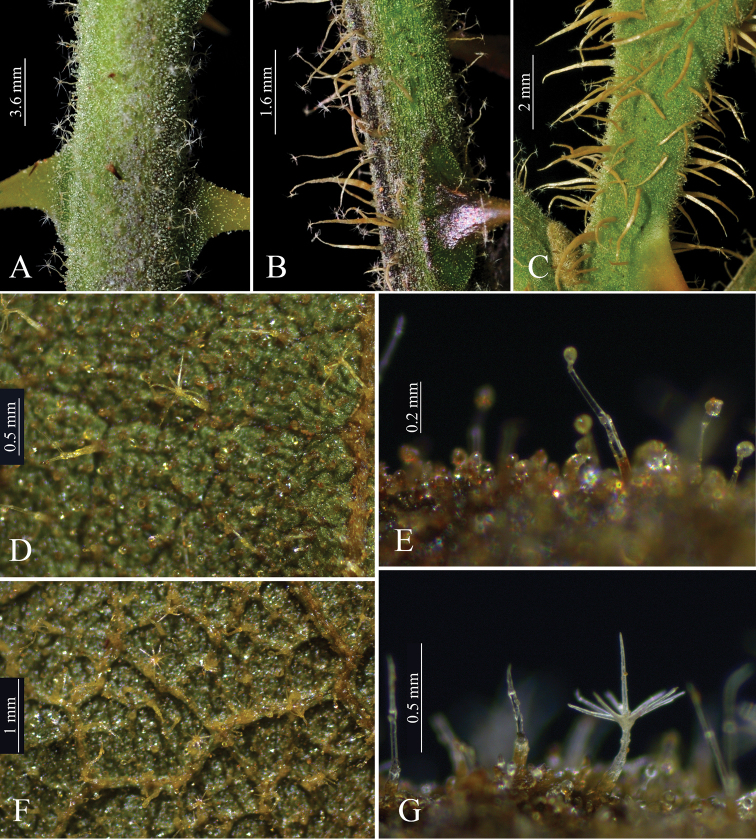
Indumentum of *Solanummedusae*. **A–C** Variation in young stem indumentum (A: *Y.F. Gouvêa* 230; B: *Y.F. Gouvêa* 264; C: *Y.F. Gouvêa* 262, BHCB) **D** Adaxial leaf surface epidermis and indumentum **E** Detail of the simple glandular trichomes of the adaxial surface **F** Abaxial leaf surface epidermis and indumentum **G** Detail of the abaxial surface trichome types (**D–G***Y.F. Gouvêa 230*, BHCB). Photographs by Y.F. Gouvêa.

**Figure 4.**
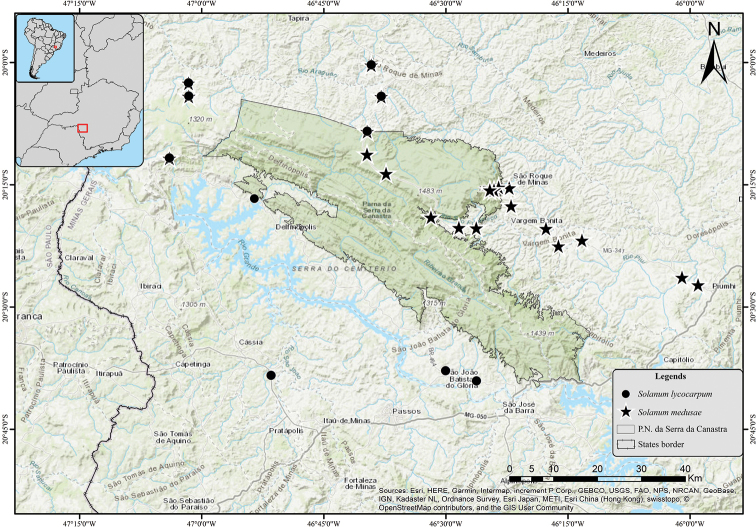
Distribution of *Solanummedusae*.

**Figure 5.**
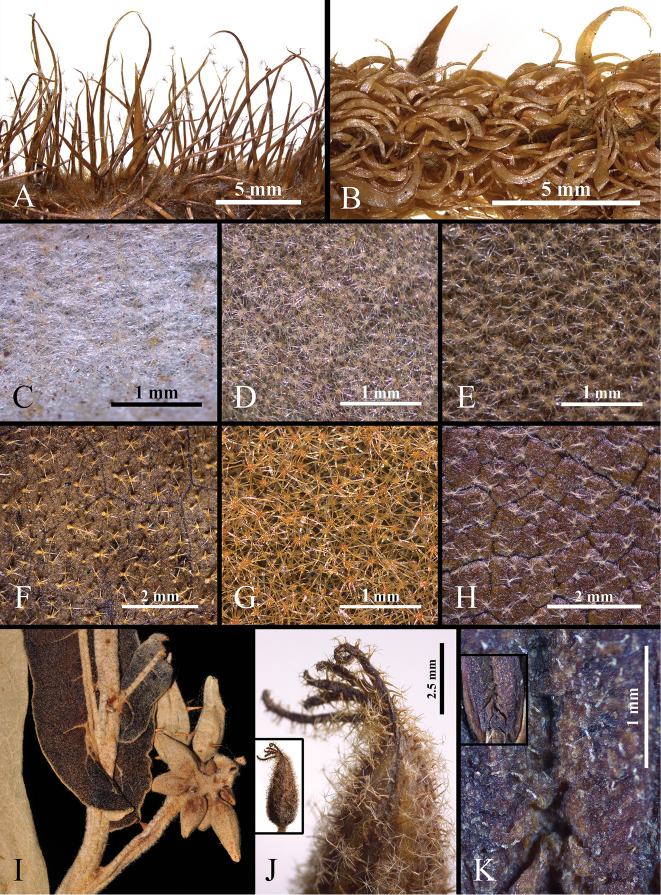
Distinctive characters of extra-Amazonian species of the Androceras/Crinitum clade. **A** Stem indumentum of *S.crinitum*; note the straight bristly stellate trichomes (*Y.F. Gouvêa et al. 196*, BHCB) **B** Stem indumentum of *S.falciforme*; note the falcate stellate trichomes (*L.F. Souza 481*, BHCB) **C** Stem indumentum of *S.lycocarpum* (*Y.F. Gouvêa 268*, BHCB) **D** Adaxial leaf surface indumentum of *S.lycocarpum* (*Y.F. Gouvêa 268*, BHCB) **E** Adaxial leaf surface indumentum of *S.falciforme* (*L.F. Souza 481*, BHCB) **F** Adaxial leaf surface indumentum of *S.quaesitum* (*U.M. Resende & V.F. Kinupp 1817*, BHCB) **G** Adaxial leaf surface indumentum of *S.crinitum* (*Y.F. Gouvêa et al. 196*, BHCB) **H** Adaxial leaf surface indumentum of *S.gomphodes* (*L.L. Giacomin et al. 1274*, BHCB) **I** Sessile sagittate leaf bases of *S.gomphodes* (*L.L. Giacomin et al. 1274*, BHCB) **J** Apiculate calyx lobe apices in *S.quaesitum*; note the extended midribs (*U.M. Resende & V.F. Kinupp 1817*, BHCB) **K** Abaxial anther surface of *S.quaesitum*; note the papillose epidermis sparsely covered by simple glandular trichomes (upper left side: detail of the distinctly swollen epidermis along the connective region; *U.M. Resende & V.F. Kinupp 1817*, BHCB). Photographs by Y.F. Gouvêa.

*Solanummedusae* is most similar to *S.lycocarpum*, the wolf-fruit, in its large berries that are yellowish green at maturity (Figure 2G), anthers with abaxially pubescent connectives (Figure 2F), curved yellow prickles, and overlap in range with *S.lycocarpum* (Fig. 4). It differs from that species in its unusual decumbent, spreading habit (Figure 2B), in the dense glandular pubescence composed of simple, uni- to multiseriate trichomes that when dry give the plant a varnished appearance (Figure 3A–G), and in its rigid, easily-broken subterranean system that grows shallow and horizontally in the soil (Figure 2C), from which new stems can emerge in some points along its length (that in some cases can be mistaken as another individual). *Solanumlycocarpum* (popularly called “lobeira”) is an iconic element of Cerrado vegetation and ecologically important for populations of the maned wolf (Portuguese: “lobo-guará”; Guarani “aguará guazú”), and is a common small tree occurring in many habitats, especially open and/or disturbed areas of the Cerrado and Caatinga biomes, and seasonally dry environments within the Atlantic forest domain in Brazil. The leaf pubescence of *S.lycocarpum* is composed of dense eglandular stellate trichomes (Figure 5D) such that the leaves usually appear densely felty and grayish green in live plants and grayish brown in herbarium specimens.

The bristly long-stalked trichomes on the young stems of some *S.medusae* (Figure 3B, C) specimens may resemble those of *S.crinitum* Lam., another widespread species of the Crinitum group in Brazil whose distribution is centered in the Amazon basin, but the presence of glandular trichomes throughout the *S.medusae* epidermis easily distinguishes it from *S.crinitum*.

*Solanummedusae* is strongly andromonoecious, with a single (to three) hermaphroditic flower at the base of the inflorescence and the more distal flowers all short-styled and functionally male (Figure 2E). Derived sexual systems like andromonoecy have arisen many times in *Solanum*, and are particularly common in the Leptostemonum Clade (Whalen and Costich 1986; Vorontsova et al. 2013). The flowers of *S.medusae* are somewhat zygomorphic with the ventral corolla lobes slightly longer than the dorsal ones and the anthers and style curved (Figure 2F); this floral form is known from species we postulate as related (Bohs et al. 2007). The stellate-pubescent abaxial connectives of *S.medusae* (Figure 2F) are also shared with *S.crinitum*, *S.falciforme* and *S.gomphodes*, which are all Brazilian endemics except for *S.crinitum*. It has been suggested (G. Davis, pers. comm.) that these act to facilitate a grip for bees buzzing the flowers, but field observations have not been undertaken to confirm this. *Solanumquaesitum*, in contrast, has the abaxial anther surface somewhat swollen (especially at the base) and papillose with sparsely distributed simple glandular trichomes (see Figure 5K); this can be used to distinguish it from other extra-Amazonian Androceras/Crinitum clade species (also see key below).

Intraspecific morphological variation (both individual and populational) of certain characters is particularly notable in spiny *Solanum* species (Roe 1966, Vorontsova and Knapp 2016, Knapp et al. 2017). In *S.medusae*, it is especially evident in indumentum, color, and prickle density of young stems. Individuals of some populations have the stem indumentum completely lacking bristly stellate trichomes (Figure 3A), whereas in specimens of other populations it is present in variable densities (see Fig. 3B, C). Young stem color ranges from completely green or partly to completely deep purple (Fig. 2D, E, 3A–C), as has been found in other *Solanum* species (e.g., *S.asterophorum* Mart., Gouvêa and Stehmann in press.). This distinct coloration can be confined to juvenile plants (as in *S.asterophorum*) or continue to be present on growing stems of reproductive individuals (as is observed in *S.medusae* and *S.kollastrum* Gouvêa & Giacomin, Gouvêa et al. 2018).

The Serra da Canastra lies in the watershed between the Paraná and São Francisco rivers. The protection of the headwaters of the São Francisco, one of the country’s most important rivers, was one of the main reasons for the establishment of the Parque Nacional da Serra da Canastra in 1972. The National Park covers about 200,000 hectares of the Cerrado biome in a landscape composed of large quartzite plateaus with areas reaching up to about 1,500 m of altitude separated by lower elevation valleys. The vegetation of the plateau highlands is formed by extensive grasslands along the flatter areas, and campos rupestres in rocky sloping areas, which especially in the Park’s northern portion gradually changes to a typical Cerrado vegetation towards valley bottoms. The region of the Serra da Canastra has a relatively long history of farming and mining, and as a result large tracts of native vegetation have been replaced by agriculture, and very few preserved areas remain outside the Park. Across Brazil, the Cerrado has one of the highest rates of deforestation, twice as fast as that of the Amazon (Klink and Machado 2005; Strassburg et al. 2017), putting species endemic to this habitat severely at risk.

We mapped the range of *S.medusae* in order to identify its limits and examine areas where it co-occurs with *S.lycocarpum* (Figure 4). Coming from the municipality of Belo Horizonte, Minas Gerais State (by the roads MG-262, MG-050, and MG-341 respectively), populations of *S.medusae* start to be found just before passing by the city of Piumhi towards São Roque de Minas (northeastern portion of Serra da Canastra), where it seems to completely replace *S.lycocarpum.* In this relatively low region where the native vegetation has been almost replaced by extensive agriculture or pastures, *S.medusae* grows on roadsides and in lesser used areas of these fields; it is not associated with the somewhat more preserved vegetation of narrow riparian forest strips. Within the Park, even with the diversity of somewhat conserved environments, *S.medusae* is mainly found in similar disturbed sites, and is less frequent as the elevation increases (from 700–800 to 1,300–1,400 m) and the vegetation changes from typical Cerrado to high elevation grasslands. In these grasslands *S.medusae* occurs as scattered individuals at road (i.e., MG-341) margins. Diverging from the MG-341 to the district of São João Batista da Serra (northern portion of Serra da Canastra), as the elevation decreases, and the vegetation gradually acquires a more typical Cerrado appearance, here *S.medusae* becomes more frequent and occurs in sympatry with *S.lycocarpum.* The two species co-occur towards the northwestern portion of the Serra da Canastra, in Cerrado areas ranging from 700 to 1,200 m elevation. In the lower (600–900 m) and relatively flat areas in the western and southeastern parts of the Serra da Canastra (i.e. municipalities of Cássia, Delfinópolis, Passos, São João Batista do Glória and São José da Barra) only *S.lycocarpum* was found.

#### Additional specimens examined (paratypes).

**Brazil**. Minas Gerais: Mun. Campinópolis, rodovia MG-341, beira de estrada, 20°21'45"S, 46°13'17"W, 729 m, May 2018 (fl), *Gouvêa 260* (BHCB [BHCB190630]); 20°22'28"S, 46°16'10"W, 807 m, May 2018 (fl), *Gouvêa 261* (BHCB [BHCB190631]). Mun. Piumhi, rodovia MG-341, beira de estrada, 20°26'19"S, 46°00'59"W, 785 m, May 2018 (fl), *Gouvêa 259* (BHCB [BHCB190629]). Mun. Sacramento, povoado de Desemboque, beira da estrada de terra que leva à MG-341, 20°02'30"S, 47°01'38"W, 1046 m, May 2018 (fl), *Gouvêa 267* (BHCB [BHCB190637]). Mun. São João Batista da Serra, saída da cidade, beira da estrada que leva de São João Batista da Serra a Tapira, 20°08'25"S, 46°39'40"W, 1150 m, May 2018 (fl), *Gouvêa 266* (BHCB [BHCB190636]). Mun. São José do Barreiro, estrada não pavimentada que leva à Cachoeira Casca d’Anta, 20°20'12"S, 46°28'24"W, 846 m, May 2018 (fl), *Gouvêa 272* (BHCB [BHCB190642]); estrada não pavimentada que leva à Cachoeira Casca d’Anta, 20°18'56"S, 46°31'50"W, 857 m, May 2018 (fl), *Gouvêa 273* (BHCB [BHCB190643]). Mun. São Roque de Minas, Parque Nacional da Serra da Canastra, primeiros trechos da principal estrada de terra que corta o PN da Serra da Canastra, 20°15'29"S, 46°24'58"W, 1283 m, 5 Apr 2017 (fl), *Gouvêa et al. 231, 232, 233* (BHCB [BHCB188230, BHCB188231, BHCB188232]); sentido P.N. da Serra da Canastra, estrada de terra que leva à “Fazenda do Chico Chagas” divergindo da estrada principal que leva à portaria 1, 20°15'16"S, 46°23'31"W, 783 m, May 2018 (fl), *Gouvêa 262* (BHCB [BHCB190632]); estrada de terra que leva ao P.N. da Serra da Canastra, beira de estrada, 20°15'36"S, 46°24'05"W, 1034, May 2018 (fl), *Gouvêa 263* (BHCB [BHCB190633]); estrada de terra que leva ao P.N. da Serra da Canastra, beira de estrada, 20°15'36"S, 46°24'36"W, 1424 m, May 2018 (fl), *Gouvêa 264* (BHCB [BHCB190634]).

### Artificial key to the Brazilian extra-Amazonian species of the *Androceras/Crinitum* clade (sensu Stern et al. 2011)

Morphological traits used to distinguish the species in the key are illustrated in Figure 5. State distributions of these species can be found on the Flora do Brasil 2020 website (http://floradobrasil.jbrj.gov.br/reflora/listaBrasil) by searching for the individual species by name.

**Table:** 

1	Decumbent shrubs; indumentum of the upper leaf surfaces composed of two layers, the longer of short- to long-stalked stellate trichomes with glandular midpoints or not, and beneath them more abundant variously sized simple glandular trichomes	*** Solanum medusae ***
–	Erect shrubs to small trees; indumentum of the upper leaf surfaces composed of a single layer of sessile to long-stalked stellate trichomes (which may seem simple because of the lack of rays, but can be recognized by their multiseriate stalks and uniseriate midpoints); simple glandular trichomes absent	**2**
2	Leaves sessile with auriculate bases	*** Solanum gomphodes ***
–	Leaves petiolate with cuneate to slightly cordate bases	**3**
3	Calyx lobe apices markedly apiculate with the midrib notably extended beyond the lobe tissue	*** Solanum quaesitum ***
–	Calyx lobe apices acute to acuminate with midrib not extending beyond the lobe tips	**4**
4	Indumentum of young stems of a single layer of stramineous sessile to long-stalked stellate trichomes with slender stalks (2–5 cells wide)	*** Solanum lycocarpum ***
–	Indumentum of young stems of two layers, the shorter sessile to long-stalked (2–5 cells wide) stellate trichomes, and the longer layer straight or falcate long-stalked bristly stellate trichomes with notably thick stalks (many cells wide)	**5**
5	Longer trichomes of young stems with straight stalks	*** Solanum crinitum ***
–	Longer trichomes of young stems with falcate stalks	*** Solanum falciforme ***

## Supplementary Material

XML Treatment for Solanum medusae
